# ‘“Health” Is Just One Piece in the Puzzle of Wellbeing’: Shifting From Preventing Health Deterioration to Improving Wellbeing in a Participatory Action Research Project With Care‐Experienced Older People

**DOI:** 10.1111/hex.70171

**Published:** 2025-02-02

**Authors:** Sarah Wallcook, Ulla Dahlkvist, Yvonne Domeij, Kerstin Green, Gigi Isaksson, Ida Goliath

**Affiliations:** ^1^ Department of Neurobiology, Care Sciences and Society Karolinska Institutet Stockholm Sweden; ^2^ Stockholm Gerontology Research Centre Stockholm Sweden; ^3^ Independent Co‐Researcher Sweden

**Keywords:** ableism, co‐research, digitalisation, effectivization, health and social care services, norms, wellbeing

## Abstract

**Introduction:**

Prevention of health deterioration is a key policy objective in Sweden informed by active and healthy ageing initiatives. However, the perspectives of older people with mobility and health limitations on current prevention initiatives are seldom gained meaning these initiatives may fail to align to with the priorities of people whose health has ostensibly already deteriorated. We aimed to explore older care‐experienced people's perspectives on the topic of health deterioration prevention and highlight aspects that they think are important to prioritise.

**Method:**

Eight older people with experience in giving or receiving formal or informal care were involved as lay co‐researchers in a participatory action research project that involved recruiting 11 further older informants to participate in peer interviews or complete a logbook. In a series of 13, 2‐h workshops held over 1 year, we undertook data generation, training, reflection and analytic activities inspired by framework analysis.

**Findings:**

The lay co‐researchers found the topic of health deterioration to be negatively and narrowly focussed opting instead to pursue a focus on articulating aspects, or puzzle pieces, that influence the improvement of wellbeing. Six influential puzzle pieces (stigma, digitalisation, services, losses, meaning and interactions) were regarded as important to prioritise which together illustrated that wellbeing is continually shaped in an interplay with dominant, but manipulable, social norms.

**Conclusion:**

This study highlights how the language of active and healthy ageing, which pervades policy and practice, is imbued with ageist and ableist subtexts that can influence older people's wellbeing and lead to exclusionary experiences in society. We highlight wider societal trends, particularly digitalisation and effectivisation, whose negative impact on older people's wellbeing could be mitigated through inclusive co‐design and resistance to normative influences.

**Public Contribution:**

This project was initiated in dialogue with stakeholder representatives from pensioner organisations in a larger scale participatory action research project. The care‐experienced lay co‐researchers collaborated in all phases of this project—gaining funding, formulating research questions, planning the study design, generating data in peer interviews, analysing and interpreting data, disseminating findings, prioritising future research and co‐authoring articles.

## 1 Background

Prevention of health deterioration is a key policy objective in Sweden, tied to the longstanding principle of ageing‐in‐place, and embedded in the Good Local Care reform and the anticipated new Social Services Law [1]. Since the 19th century, health deterioration continues to be regarded as a condition of old age and, in tandem with an apocalyptic view of the increasing ageing demography, prevention strategies aim to achieve a reduction in the economic and societal impact of this so‐called ‘meat mountain’ [2]. Within this framing, human longevity is regarded as a threat to the welfare state and inherently associated with decline, frailty and increasing costs, rather than benefits, to society [3]. However, instead of increasing as anticipated, the proportion of Sweden's older population receiving social support (i.e., home care, day centres, residential care) is observed to be reducing, a trend which began even before the Covid‐19 pandemic [4, 5].

To counter the doomsday narrative that old age incurs disability and disease, much‐needed movements such as active‐, healthy‐ and successful ageing have arisen [6]. However, an unanticipated consequence is that these movements predicate upon ableism and create the conditions for older people who have or acquire health deteriorations and disabilities to be regarded as failing to age successfully [7]. Such experiences of failure can interfere with older people's interactions with the health and social care system, excluding this important target group from the prevention agenda and leading to marginalisation in society [8].

Prevention work with older people generally focuses on decreasing and delaying health deterioration through maintaining functional ability and delaying an increased need for societal services. The work thus includes a wide spectrum of mostly individualistic, specific and social interventions across physical, mental and social wellbeing domains [9, 10]. However, with a few exceptions, holistic interventions are scarce and activities initiated by welfare services often result in time‐limited projects rather than integral approaches used in everyday work [11]. Moreover, people aged over 85, and older people with mobility and health impairments are regarded as harder to reach [12, 13] which risks overlooking their perspectives on prevention initiatives. Indeed reviews of interventions that aim to prevent frailty and falls have noted that the goals of older people already in situations of frailty are not always addressed [14, 15].

This knowledge gap around whether initiatives match older people's diverse needs could ultimately undermine societal services' ability to reach their intended target group and achieve health deterioration prevention outcomes. Developing this knowledge calls for listening to older people's health priorities, including those people who are seldom heard, and incorporating their perspectives in prevention work. The scant studies that have done so point towards a need to go beyond biopsychological priorities and consider faith, autonomy, service provision and societal pressure as relevant to incorporate. These studies however are mostly contextually specific with only one based in Sweden, and people with disabilities and care needs are poorly represented [16, 17, 18]. Consequently, our study aimed to explore older care‐experienced people's perspectives on the topic of health deterioration prevention and highlight aspects that they think are important to prioritise.

## 2 Methods

**Figure 1:**
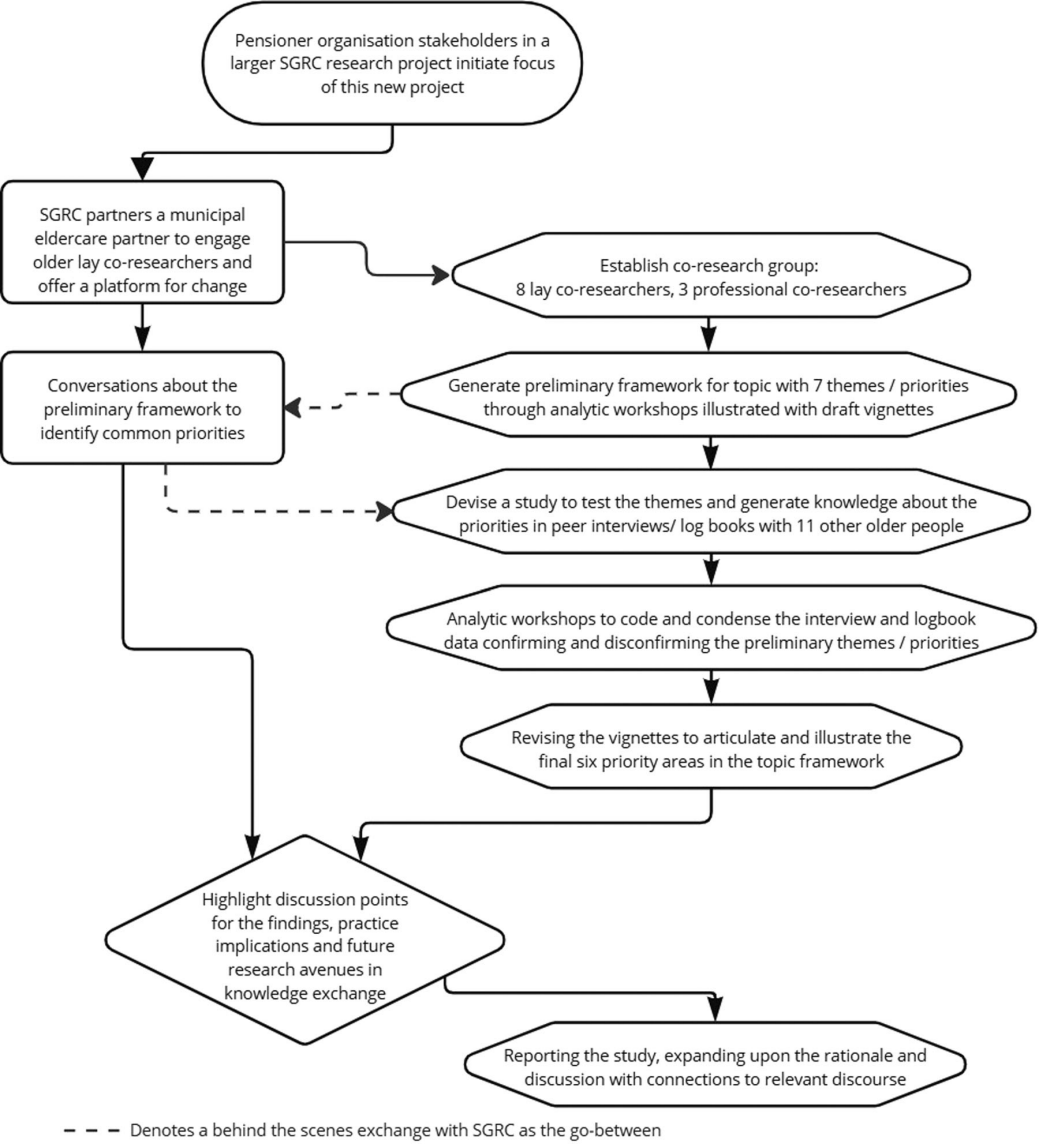
Overview of our PAR approach.

The Stockholm Gerontology Research Centre (SGRC) is a research and development unit that provides practice‐based research and organisational development support to health and social care services. This Participatory Action Research (PAR) project was initiated from feedback provided by stakeholder representatives from pensioner organisations in SGRC's larger scale PAR project, SAMSAS, co‐creating collaborative models to prevent health deterioration. As members of SAMSAS' reference group, these representatives' feedback highlighted that researchers needed to go beyond current stakeholder involvement to more broadly explore older people's perspectives on health prevention deterioration. From this starting point, we planned a PAR project that involved and engaged lay older people across the research cycle as co‐researchers. In this approach, we could respect older people's expertise in their own situation and build upon collective enquiry and reflection to understand and co‐research the issues that were important to them [19]. Figure 1 summarises our approach, which is described and critically reflected upon in detail in a separate article [20].

### 2.1 Older People's Involvement and Engagement

SGRC partnered with a local prevention unit in municipal eldercare to involve eight people aged 78–92 as lay co‐researchers (LCRs) to collaborate equitably in all phases of the research project. Rather than calling upon their existing pool of service users, eldercare partners reached out to the wider community via an advertisement in the free local newspaper and posters in municipal venues and meeting places for older people. Formal inclusion criteria were that LCRs were aged 75 and over and residing within the specific city district of the prevention unit's jurisdiction. Beyond this, the lived expertise that partners sought among LCRs was their experience of either giving or receiving informal or formal care, which could indicate insider knowledge of living with health deterioration and navigating care systems. Moreover, the advertisement stipulated that the project was about health deterioration prevention and that adjustments would be made to support people with physical, memory and cognitive impairments to become involved, with significant others also welcome. LCRs were free to decide the duration of their involvement over this 1‐year project with 13 in‐person fortnightly 2‐h long workshops led by three professional co‐researchers (PCRs) from SGRC (including S.W. and I.G.) LCRs' roles were tailored to their strengths and interests and considered equal to those of PCRs [21]. After six initial voluntary workshops LCRs received a fee per task they were involved in and four LCRs (co‐authors U.D., Y.D., K.G., G.I.) remained involved until the end of the project. These four LCRs have subsequently been involved in communicating the results of this study and their presentations to different audiences form the central support to how this manuscript has been written. S.W. has been responsible for drafting the English text in this report, staying as close as possible to our oral presentations. However, the writing process necessitates further expansion upon our rationale for the study and discussion of the results and implications. To maintain involvement through this writing process, S.W. provided summaries and translations in Swedish, highlighting where expansions have occurred and encouraging further discussion before agreeing the final text.

### 2.2 Study Design and Planning

Our qualitative study design focussed on an often neglected area of involvement in research, namely formulating research questions and issues [22, 23]. Consequently, we addressed one PAR building block of establishing a common understanding of the of the issue at stake [21] by defining and redefining the problem of health deterioration prevention. To produce a common understanding of the problem, LCRs generated and collaboratively analysed data with PCRs in a series of reflective PAR cycles that gradually expanded relationships and deepened our understanding of the issue [24]. These cycles were conducted in a three‐phase process over 1 year, (i) the group working alone to establish collective knowledge, (ii) reaching out more widely to test that knowledge, (iii) exchanging knowledge in events with multi‐sector actors [20].

### 2.3 Ethics

The Stockholm Regional Ethical Committee granted permission for this study (Diary numbers 2021‐04645, 2022‐02744‐02), which included engaging older people as LCRs to generate data with other stakeholders through methods such as interviews and focus groups. Written informed consent was taken from LCRs and participants, who were all reminded of their right to withdraw from the study at any time and without giving reason.

### 2.4 Data Generation

In a series of six audio‐recorded, photographed and annotated workshops with a mix of group and individual activities, LCRs generated data by pooling their lived experiences on the topic to arrive a preliminary definition of the problem. Workshop planning was agile with each workshop addressing a twofold aim, firstly to generate data relevant to the enquiry, secondly to generate curiosity and questions that informed planning for the next workshop. This process unfolded from the first workshop's individual and joint activities, which stimulated sharing personal connections to the topic of health deterioration and prevention. Ultimately, this first phase of the project culminated in the question of how LCRs could attain greater trustworthiness in their preliminary problem definition. A detailed description of these workshop procedures and outputs is provided in a separate article [20]. In seven further workshops with training, reflective and analytic activities, LCRs then co‐designed and undertook a qualitative enquiry with PCRs that would facilitate further exploration of the problem and enhance the credibility and transferability of the preliminary definition. In this enquiry, data were generated by conducting semi‐structured audio‐recorded peer interviews or gathering logbooks with other older people and holding post‐data generation reflective discussions in pairs and as a group. The interviews, logbooks and reflective discussions were supported by guides co‐written by LCRs and PCRs [20]. A questionnaire was used to gather demographic information that the co‐research team regarded as relevant for describing the participants in relation to the study focus. In this questionnaire, information about race and ethnicity was not gathered as such practices are moreorless prevented in Sweden due to prevailing norms of colourblindness and antiracialism [25].

### 2.5 Participants and Recruitment

LCRs purposefully invited friends and acquaintances from their networks to participate in the study on the assumption that, in common with LCRs, the prospective participants would share an interest in the topic and be willing to give information rich accounts from varied perspectives [26]. This non‐probabilistic, purposeful snowball sampling approach, is particularly suited to this study as it facilitates access to, and focus on the applicability of, participants' lived expertise to the topic [26]. Eligible participants were aged 75 or over, with experience of giving or receiving informal or formal care, living in the community in ordinary or supported housing (i.e., not a care or nursing home) and able to take part in Swedish in an interview at home or another place of their choosing, or via writing a logbook. We assessed the concept of information power when considering our sample size [27]. We reasoned that the LCRs had already narrowed the focus of the study and sharpened the theoretical perspectives on the problem through their six workshops. Moreover, given that LCRs were already engaged in the topic, we anticipated that snowball recruitment through their networks would lead to specificity in the sample, that is participants who belong to the target group exhibiting varied perspectives. However, the LCRs themselves were mostly unfamiliar with holding research interviews so that tailored support was offered from the PCRs to strengthen the quality of the dialogue and each LCR planned to conduct at least two interviews [20]. In light of this assessment, we regarded that 8–13 interview participants would be sufficient to explore patterns across the data and confirm or disconfirm the problem that LCRs had preliminarily defined. Eleven people opted to take part in the study, 10 as peer‐interviewees and 1 contributed a written logbook (for participant characteristics see Table 1). Participants, except for two, chose to be interviewed by an LCR who was unknown to them.

**Table 1:** Characteristics of participants who took part in the co‐designed qualitative enquiry.

Participant characteristics	*N* = 11
Gender	
Woman	10
Man	1
Age	
75–79	3
80–84	4
85–89	2
90–94	2
Highest level of education	
Primary	3
Secondary < 3 years	0
Secondary ≥ 3 years	0
Vocational college or university < 3 years	2
University ≥ 3 years	6
Working life	
Service industry	3
Health and social care	5
Education	2
Information and media	1
Self‐rated general health	
Excellent	0
Very good	4
Good	0
Passable	5
Poor	2
Health conditions and impairments^a^	
No diagnosis or impairment	3
Medical diagnosis	2
One impairment	1
Medical diagnosis, one impairment	4
Two impairments	0
Medical diagnosis, two impairments	1
Living situation	
Co‐habiting	3
Living alone	8
Self‐rated participation outside home	
I participate the way I wish	3
I almost participate the way I wish	6
I do not participate as much I wish	2
I do not at all participate as I wish	0

^a^ Impairments include sensory, cognitive, mobility, motor.

### 2.6 Analysis

PCRs and LCRs collaboratively and iteratively analysed the data through reflective activities and discussions as the data was being generated [24]. Within the workshops, written notes were collated and sorted in a large, interactive visual format in which everyone contributed to the placing, sorting, re‐sorting and grouping of data to create preliminary categories. Author S.W. listened repeatedly to workshop audio recordings, took further notes, wrote reflexive journals and had analytic discussions with the other PCRs between each workshop. Summaries of the process from and between each workshop were presented back in the next workshop and discussed to maintain the whole team's familiarity with the data and analytic progress [20]. Our approach to categorising LCRs' workshop data was inspired by the procedures described in framework analysis, which we used to inductively produce a set of preliminary themes for the problem definition [28]. These themes were then deductively confirmed and disconfirmed by comparing them with participants' interview and logbook data, which enriched and deepened our analysis and knowledge of the problem [26].

To retain, but make manageable, the richness of our expanding data set, the PCRs worked with recordings and transcripts to produce vignettes for each category that LCRs discussed and reworked sequentially [20]. Vignettes are short stories that distil complex and sensitive individual and group experiences and function as a tool for reflective and analytic dialogue about norms and values [29]. Within this dialogue, LCRs and PCRs explored subjectivity and unpacked and confronted their personal biases when juxtaposing participants' and LCRs' accounts. The final version of each theme's vignette captures the exchange between LCRs in their workshops and with peer interviewees and is featured in our illustration of the findings.

Our final stage of analysis took place in knowledge exchange seminars with our eldercare partners who confirmed the familiarity of the themes, which we consider further enhances the credibility and transferability of the problem definition. Our partners' input further led to a dynamic exchange of ideas and expertise that contributed to how we situated and prioritised our defined problem within eldercare policy and practice [30].

## 3 Findings

### 3.1 The Shift From Health Deterioration Prevention to Improving Wellbeing

Early in the process, the group highlighted the deficit and bio‐medical focus of the ‘health deterioration prevention' language. This language was described as creating a negative feeling to the conversation, and that the focus on health was too narrow. Health according to LCRs, was restricted to pathologies within the body, a view which was reinforced within the narrow scope of the healthcare professions that LCRs encountered. As such, there was a consensus that the possibility to control and influence one's own health status was limited at this stage in life, since incidences of poor health were not something a person chooses. Consequently, LCRs considered the conversation of health deterioration prevention to be alienating and exclusionary to older people who perceived their health to be viewable by others, or subjectively, as poor.

Conversely, LCRs identified the salience of a wider focus on ‘improving wellbeing’ which they considered to be semantically salutogenic, i.e. more focussed on assets than deficits. As a term, wellbeing provided greater scope for a positive and broad view that encompassed aspects outside the body and the immediate sphere of an individual's influence. Moreover, LCRs chose ‘improving’ as the optimal term since ‘promoting’ was felt to be too passive, and ‘maintaining’ or ‘sustaining’ implied that one must already experience wellbeing to an acceptable degree. It followed that LCRs regarded individual wellbeing as possible to influence and improve at multiple levels, specifically policy and media, health and care systems, community and individual. With wellbeing in focus, the project moved on to elucidating influential aspects of wellbeing that are important to prioritise in research and practice.

### 3.2 The Prioritised Puzzle Pieces That Influence Wellbeing

**Figure 2:**
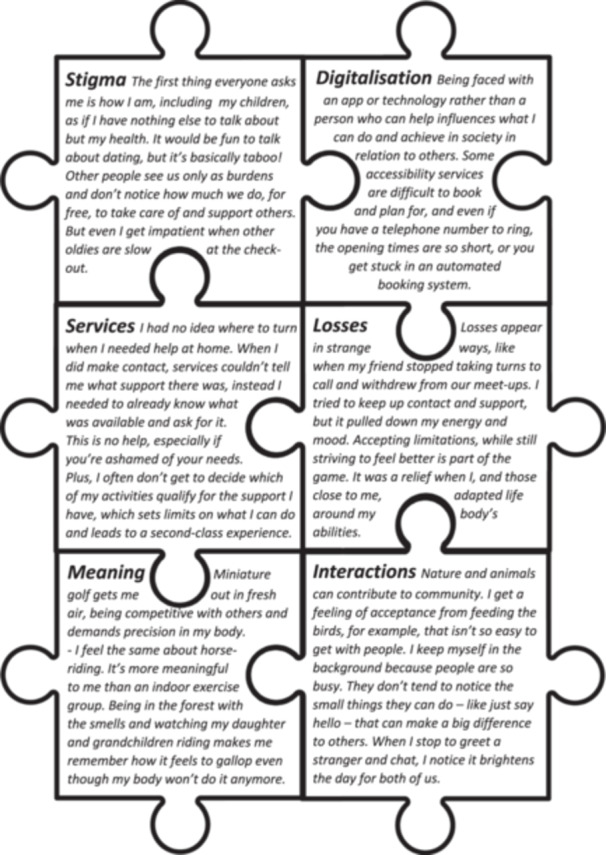
Vignettes illustrating the six key puzzle pieces of wellbeing that were constructed through our co‐research.

LCRs regarded that ‘health is just a small piece in the puzzle of wellbeing’, and that rather than gaining its own separate puzzle piece, health interacted as inequalities connected to the avoidable and unfair distribution of wealth and opportunity between different groups in society. Fundamentally, our co‐research understands that wellbeing is not dependent on good health, but health may be positively influenced, and influenceable, by wellbeing. In our study, wellbeing came to be regarded as ‘Something of a package. It involves more of a feeling perhaps that one's overall life situation is okay’. The six key pieces of our puzzle, that our co‐research explored as key influencers of wellbeing are illustrated by the vignettes (created through the analytic process) contained in Figure 2. This puzzle contains no edge pieces to convey that these priorities are not intended to comprise an exhaustive account of aspects that influence wellbeing. Neither are these six prioritised puzzle pieces hierarchised as relatively more or less important influencers of wellbeing in relation to each other.

The vignette on the puzzle piece, stigma communicates the situations that arise when an older person's self‐concept is not upheld in encounters with other people. In such situations, the lack of match between self‐perception and others' perception means the older person feels unrecognisable to themselves, in terms of who they are and how they contribute, by having been unrecognised by others. An implication of this puzzle piece is that wellbeing can be positively influenced by seeing beyond age to be non‐judgemental in interactions with older people and break down ageism.

Digitalisation describes how digitalised services, both public and private, are designed in ways that contribute to whether older people feel part of an equal and just society. Such services determine whether the concessions and provisions made are sufficient to facilitate participation leaving little scope for individual autonomy and control. Yet, exclusionary encounters with digitalised services negatively impact on participation in society and daily life. The implication here is that services in society can support wellbeing when digitalisation is experienced as accessible, useable and inclusive.

Leading on, services focuses on eldercare services and the helplessness and shame that can be experienced with navigating this system. This is juxtaposed with the perception that the services offered by the system are being gatekept so that pre‐knowledge of the right thing to say or do is needed to access support. The implication is that easily understandable information about services, provided in accessible formats, as well as enabling services to be used according to preferences, enhances wellbeing among older people who need and have a right to support.

Losses take a nuanced view of grief and loss to spotlight the relational aspects of sequential internal and external changes and the irreplaceability of old friends with new acquaintances. Grieving for and adjusting to these many and continual losses and changes can challenge older people's wellbeing whilst providing opportunities for learning and personal growth. The implication here is that older people's wellbeing is impacted by the need to continually negotiate and adapt to the changes going on both within themselves and those around them.

Meaning points to how wellbeing connects to upholding personal identity that is a person being able to continue being who they are, by doing what matters to them. This may mean that while activities are adapted, they are still intact with the way they have always been done, providing a sense of continuity concerning identity and/or intergenerational connection. The implication is that people not only need to be able to do personally meaningful activities, but also that they should be able to do them in ways that are personally meaningful.

The final puzzle piece, interactions acknowledges the reciprocity and sense of community derived in small exchanges with both human and nonhuman actors. Within these interactions, older people may achieve satisfaction from actively contributing towards creating a better and more friendly society that provides for everyone. The implication of this puzzle piece is to resist the pressure to be productive and effective, and put more emphasis on noticing the small things that people do that can make a big difference in wellbeing for all.

#### 3.2.1 A Picture Illuminated by the Pieces

The puzzle pieces are unified as a set of transformative and dynamic internal and external forces that interact with wellbeing. In this view, wellbeing is continually shaped in an interplay with dominant social norms, for example, ageist and ableist assumptions, ideals of positivity and resilience, expectations of conformity, productivity and effectiveness. These norms, when deviated from, can destabilise wellbeing and what it is to feel okay about one's life situation. Revealing this picture illuminates the futility of individualising the issue of improving wellbeing. Instead, the picture articulates the need to dismantle and resist the normative influences that operate at multiple scales to limit and oppress wellbeing.

## 4 Discussion

In our co‐research, we explored older care‐experienced people's perspectives on the prevention of deterioration of health. Our key finding was that the older people involved in our project would like to replace the exclusionary language of health deterioration prevention, which pervades eldercare policy and practice in Sweden. They proposed instead a focus on improving wellbeing, and subsequently articulated six puzzle pieces that they regarded as key influencers of wellbeing. This shift in study focus thrust our co‐research group into new territories, which not only shaped what we did, but also led to a repositioning within the background discourses that motivated our study. We first discuss how our findings contribute to the knowledge of wellbeing and the merits and drawbacks of our approach. We conclude our discussion by considering wellbeing in relation to the language used in health and social care policy and practice centred on older people.

### 4.1 Wellbeing in Older Age and This Study's Contributions to the Field

Wellbeing is an extensive body of research that has attracted increased interest over the last 10 years [31]. This interest includes specific focus on later life, where it has been argued as relevant to identify the factors that may be conducive or detrimental to wellbeing [32]. Our findings contribute to this call by highlighting the influence of manipulable norms across six wellbeing themes that traverse individual, social and institutional levels. In calling to resist these norms, our study agrees with previous co‐research with older people that similarly argued against wellbeing as an individual responsibility and pointed towards wellbeing as a relational and collective concept [33]. In our study, we also acknowledge the tension that co‐exists where resisting norms can lead to norms being reinforced. For example, resisting stereotypes of old age dependence and disability can simultaneously contribute to devaluing and discrediting older and/or disabled people in society perpetuating ageism and ableism [34].

Our construction of wellbeing aspects that are important to consider overlaps with studies that similarly draw attention to the influence of digitalisation on wellbeing and the importance of accessible and inclusive societal services [35, 36, 37]. Identifying these systemic influences on individual wellbeing is significant as service providers face effectivisation pressures that influence the design and delivery of their initiatives, with or without digitalisation [38]. By service providers, we mean any public and private sector providers whose services are intended for use by older people. This encompasses, and is not limited to, regional and municipal services (i.e., specialist and general healthcare appointments, accessibility services such as writing and sign language interpreters, eldercare in the community, disability travel service). However, probable influencers of wellbeing tend to be understood in terms of individual factors related to age [39], activity [40, 41, 42] and wider socioeconomic inequalities [43]. Moreover, societal transformations, such as digitalisation, and the effectivisation of health and social services initiatives have a complex interplay with wellbeing. In this interplay, digitalisation and modes of service provision may be infrastructurally predisposed to having a detrimental impact on wellbeing at the same time as positive contributions to wellbeing can co‐exist [44, 45, 46, 47]. Service providers therefore have an opportunity to strive for inclusive design that mitigates the potential negative impacts that digitalisation and effectivisation (particularly the two in combination) could have on the wellbeing of older people.

### 4.2 Merits and Drawbacks of Our Approach

Partnerships in PAR are vital and our approach took the unusual step of working more distantly with our municipal eldercare partners in the first instance and building a closer collaboration over time. While this step was advantageous in enabling us to elucidate a more complete picture of older people's perspectives, we acknowledge that this came at the cost of taking action together with our partners [20]. However, understanding this project as a seed for change and future service development, we identified overlaps between aspects of wellbeing that both LCRs and municipal partners have a shared stake in investigating and addressing. These aspects will be attended to in new cycles of PAR in a funded 6‐year programme that will co‐create models, tools and innovations for inclusive homecare services that foster older people's wellbeing.

Our way of overcoming resistance to reporting on ethnicity and race in Sweden is to disclose that everyone involved in this research visibly passed as Swedish. We perceive that this lack of representation hampers the transferability of our findings as older people who may experience compounding disadvantage due to racial or ethnic discrimination have not been active in the construction of this knowledge [13]. Moreover, our findings predominantly feature the perspectives of women rather than men, which may be regarded as constraining the utility of our study particularly in light of known gender inequalities in wellbeing [48]. However, that women opted to become involved in our study is not surprising given the dominance of women in both formal and informal care work [49] and our original entry point as preventing health deterioration. Consequently, we assert the value of gaining these women's perspectives and experiences of wellbeing which they accrued through a lifetime of engagement in caring. It is precisely LCRs engagement in informal and formal care which was central to recruitment and purposefully accessing the information rich perspectives of older people who may be regarded as hard to reach due to isolation at home [26]. This means that the interview took place in a space of shared lived experience that disrupted interviewer neutrality, enhanced the humanity of the conversation and offered the possibility of mutual self‐disclosure [50]. However, this step in our process meant that LCRs recruited people known to them which entails risks to, among things, personal integrity and power within these relationships [50]. Risks are only magnified if the LCR is also involved in the peer interview with their acquaintance or friend [51]. To explore these risks and make a principled decision about who would interview whom, as part of the training, we engaged in co‐reflection within our co‐research team to explore the possible benefits and burdens in each case. Equipped with a more detailed understanding, LCRs (if they had not already ruled themselves out as the interviewer) took this discussion to their recruited acquaintance or friend. They then decided together which option felt more appropriate within the bounds of their own relationship. Ultimately, only one LCR interviewed people known to her and we noted that these relationships shared three unique characteristics: The presence of informal care‐giving, familiarity with the informant's situation and the informant's preference for having that LCR's support to host the interview at home [20].

### 4.3 Wellbeing and the Language of Health and Social Care Practice and Policy in Sweden

Sweden, together with Denmark, Finland, Iceland and Norway are regarded as comprising the Nordic welfare model, which faces financial challenges to the underpinning principle of universalism (i.e., public service provision based on needs, not means) [52]. Within this Nordic context, a recent policy analysis in Norway demonstrated how the ageist and ableist norms within the successful and healthy ageing agendas were embedded in current policy and shaped expectations for older people [38]. In our study, the policy framing of ‘prevention’ and ‘deterioration’ was seen as alienating to people who already have health concerns, that is they have failed to prevent their own unsuccessful ageing [53]. Crucially our findings highlight that word choices in eldercare policy are not neutral and in agreement with a narrative analysis of government policy documents, we similarly see that these policy articulations are informed by and perpetuate norms [54]. Consequently, we argue that word choices have an impact on the way that older people then go on to experience their interactions with the services that are shaped by these policies. Service providers' awareness of the impact of language to reinforce and/or resist normative influences can promote more inclusive communication that supports equitable service uptake among older people. Indeed, this point about word choices inspired discussions about straightforward ideas for change among stakeholder service providers who were involved in our knowledge exchange activities [20].

## 5 Conclusion

This study, designed and undertaken with older care‐experienced co‐researchers highlighted a preference for focus on improving wellbeing, rather than seeking to prevent health deterioration. This clearly exemplifies a lynchpin motivator for involving people in research who have a stake in the generation of that knowledge. Namely, that such involvement can lead to a lightbulb moment between stakeholders where irrelevant and even repellant study objectives materialise and can be addressed. In our study, the LCRs' subversion of our original study purpose illuminates how the active ageing language imbued in eldercare policy and practice can resonate poorly and lead to a mismatch between the priorities of services and the priorities of the target user group. This poor resonance arises from the ageist and ableist subtexts that can inadvertently discriminate against and alienate older and/or disabled people. Moreover, rather than individualising responsibility for wellbeing, our study highlights the influence of wider societal trends, such as digitalisation and effectivisation of services, which could be mitigated through inclusive co‐design. This study thus articulates a need to resist the normative influences in society that operate at multiple levels to negatively impact upon older people's wellbeing.

## Author Contributions

**Sarah Wallcook:** conceptualisation, funding acquisition, writing–original draft, investigation, methodology, writing–review and editing, formal analysis, project administration, data curation, resources. **Ulla Dahlkvist:** conceptualisation, investigation, methodology, formal analysis. **Yvonne Domeij:** conceptualisation, investigation, methodology, formal analysis. **Kerstin Green:** conceptualisation, investigation, methodology, formal analysis. **Gigi Isaksson:** conceptualisation, investigation, methodology, formal analysis. **Ida Goliath:** conceptualisation, investigation, funding acquisition, writing–original draft, methodology, writing–review and editing, formal analysis, supervision.

## Ethics Statement

The Stockholm Regional Ethical Committee granted permission for this study (Diary numbers 2021‐04645, 2022‐02744‐02).

## Consent

All co‐researchers and participants gave their written informed consent to be involved in this study.

## Conflicts of Interest

The Stockholm Gerontology Research Centre's board comprises elected politicians appointed by Stockholm's regional and district councils. Beyond this contextual detail, the authors have no conflicting interests to disclose.

## Data Availability

The datasets generated and analysed during the current study are not publicly available due to the risk of compromising the co‐researchers’ integrity and ownership of the findings.
